# Screening a DNA Aptamer Specifically Targeting Integrin β3 and Partially Inhibiting Tumor Cell Migration

**DOI:** 10.1021/acs.analchem.3c01995

**Published:** 2023-08-09

**Authors:** Xiaoyan Teng, Yu Wang, Liuxia You, Lirong Wei, Chao Zhang, Yuzhen Du

**Affiliations:** † Department of Laboratory Medicine, Shanghai Jiao Tong University Affiliated Sixth People’s Hospital, Shanghai 200233, China; ‡ State Key Laboratory of Oncogenes and Related Genes, Shanghai Cancer Institute, Department of Oncology, Institute of Molecular Medicine, Renji Hospital, School of Medicine, Shanghai Jiao Tong University, Shanghai 200127, China; § Department of Clinical Laboratory, The Second Affiliated Hospital of Fujian Medical University, Quanzhou, Fujian 362000, China

## Abstract

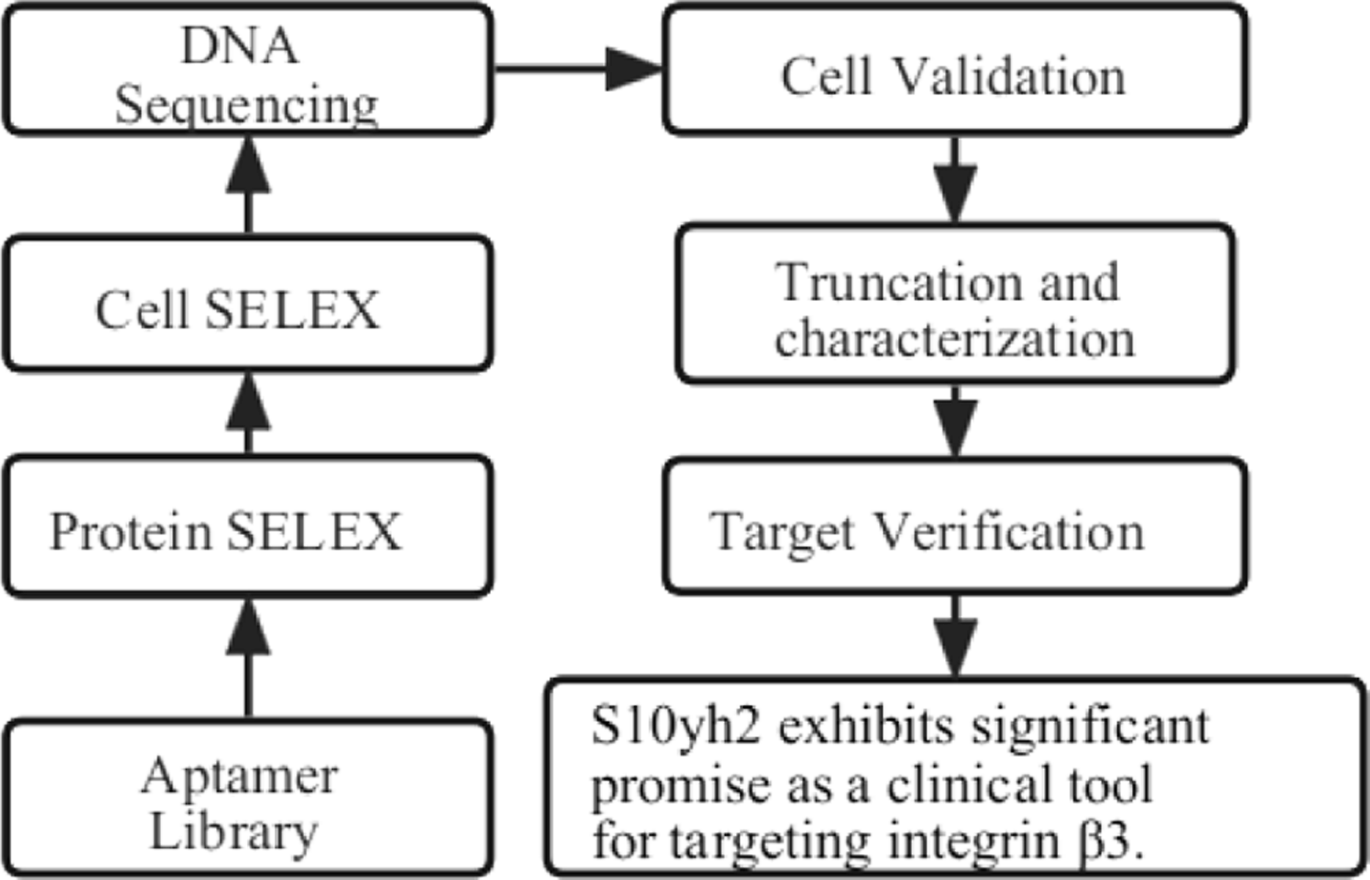

Due to its key roles in malignant tumor progression and reprograming of the tumor microenvironment, integrin β3 has attracted great attention as a new target for tumor therapy. However, the structure–function relationship of integrins β3 remains incompletely understood, leading to the shortage of specific and effective targeting probes. This work uses a purified extracellular domain of integrin β3 and integrin β3-positive cells to screen aptamers, specifically targeting integrin β3 in the native conformation on live cells through the SELEX approach. Following meticulous truncation and characterization of the initial aptamer candidates, the optimized aptamer S10yh2 was produced, exhibiting a low equilibrium dissociation constant (*K*_d_) in the nanomolar range. S10yh2 displays specific recognition of cancer cells with varying levels of integrin β3 expression and demonstrates favorable stability in serum. Subsequent analysis of docking sites revealed that S10yh2 binds to the seven amino acid residues located in the core region of integrin β3. The S10yh2 aptamer can downregulate the level of integrin heterodimer αvβ3 on integrin β3 overexpressed cancer cells and partially inhibit cell migration behavior. In summary, S10yh2 is a promising probe with a small size, simple synthesis, good stability, high binding affinity, and selectivity. It therefore holds great potential for investigating the structure–function relationship of integrins.

## 1 Introduction

Cancer is a complex disease that poses a threat to human health. Multiple factors influence malignant tumors, including signaling pathways that promote angiogenesis, metastasis, and invasion, energy metabolism reprograming, and immune surveillance evasion.^1^ Increasing evidence emphasizes the importance of the tumor microenvironment, and regulation of the tumor microenvironment is considered a promising cancer treatment strategy.^1^ Consequently, screening for multifunctional targets closely associated with malignant tumors and reprograming the tumor microenvironment are imperative.

Integrin β3, also known as CD61 or GP3A, is a vital member of the integrin family. Integrin β3 is involved in tumor metabolism, extracellular matrix remodeling, shaping of the immune microenvironment, and promoting epithelial–mesenchymal transition, indicating its crucial role in the malignant progression of tumors and the reprograming of the tumor microenvironment.^2^ The abnormal expression or upregulation of integrin β3 is crucial in the pathogenesis of various solid tumors, including lung, prostate, and breast cancer. Overexpression of integrin β3 plays a significant role in tumor cell proliferation, angiogenesis, tumor invasion, and metastasis. Integrin β3 forms heterodimers with αIIb and αv, selectively binding to ligands containing active RGD peptides.^3^ High expression of αIIbβ3 integrin in platelets is associated with the pathogenesis of Glanzmann thrombasthenia and plays an essential role in the occurrence and development of platelet tumors.^4,5^ Upregulation of αvβ3 integrin in endothelial cells and tumor cells promotes invasion and migration of various malignant tumors.^2,6,7^ Consequently, integrin β3 has been identified as an important prognostic factor associated with low survival rates in various cancers.^6,8,9^ In general, integrin β3 may have broad prospects as a new target closely related to the tumor microenvironment.

In clinical research, most detection and treatment strategies targeting integrin β3 rely on anti-integrin β3 peptides or antibodies. Currently, peptidic drugs targeting integrin β3 used in clinical research and anti-integrin β3 antibodies used in clinical research, such as cilengitide and vitaxin, have certain limitations, including large size, high instability, difficulties in chemical modification, and strong immunogenicity.^10^ The mechanism by which cilengitide operates entails the inhibition of tumor angiogenesis and the suppression of tumor cell adhesion and migration, thereby exerting its anti-tumor properties. Nevertheless, clinical trials have demonstrated that cilengitide did not attain the anticipated efficacy in specific cancer treatment modalities. The lack of success of cilengitide in the field of oncology underscores the intricate nature of tumors and the existence of evasion mechanisms. Tumor cells frequently exhibit heterogeneity and evasion strategies, enabling them to circumvent the impact of cilengitide through diverse pathways. This may encompass the activation of alternative signaling pathways, the occurrence of mutations, and the presence of tumor cell heterogeneity, alongside other contributing factors.

The sensitivity to environmental conditions (i.e., temperature and pH) also limits their widespread applications in cell labeling and imaging. Small molecule inhibitors such as MK-0429, although exhibiting high affinity to the purified αvβ3 heterodimers, showed lower target specificity and may have limitations for in vivo stability and efficacy.^11^ Consequently, the identification and management of cancer require a more stable and efficacious integrin β3 binding ligand that not only surpasses conventional molecule inhibitors but also exhibits distinctive properties. The significance of the β3 subunit in tumor development and metastasis is acknowledged. This subunit plays a crucial role in tumor angiogenesis, platelet activation, and tumor cell adhesion. Consequently, the screening and development of aptamers that specifically target the β3 subunit hold potential for intervening in these processes and yielding favorable outcomes in the field of oncology treatment.

Aptamers, as short single-stranded oligonucleotides composed of either DNA or RNA, exhibit remarkable affinity and selectivity toward a diverse array of targets, ranging from small molecules and ions to complex mixtures like cells.^12,13^ The interaction between aptamers and their targets relies predominantly on structural compatibility, aromatic ring stacking, electrostatic and van der Waals interactions, hydrogen bonding, or a combination of these mechanisms.^14,15^ Functionally, aptamers share similarities with protein antibodies. However, due to their unique oligonucleotide properties, aptamers possess distinctive chemical and biological characteristics that differentiate them from traditional antibodies. These characteristics include facile chemical synthesis, low molecular weight, high chemical stability, absence of immunogenicity, low toxicity, rapid tissue penetration, and versatility regarding modification and manipulation.^16^ As a result, aptamers hold immense potential in various aspects of cancer research, including cancer diagnosis, biomarker discovery, treatment, and drug delivery.^17,18^

A previous study has reported an RNA aptamer targeting integrin β3. However, it only validated the application of this RNA aptamer in ELISA detection in vitro and did not show data for binding to the intact integrin β3 on live cell membranes.^19^ Compared with RNA aptamers, DNA aptamers have advantages such as higher stability and easier synthesis.^20,21^ Currently, there are no reports of DNA aptamers targeting integrin β3, especially for the integrin β3 on cell membranes.^22^ The reasons include difficulties in obtaining intact membrane proteins of integrin β3 as the SELEX target and a short systematic method for the in situ selection of specific membrane proteins under a complex cellular environment.^23^

Several integrins, including αvβ3, αvβ5, and αvβ1, have been investigated in SELEX studies. Specifically, avb3 has been extensively examined. The current study used a combination of in vitro SELEX and cell-based SELEX techniques to identify DNA aptamers that specifically bind to integrin β3 with high affinity. Among the selected aptamer candidates, the optimal aptamer candidate (S10yh2) specifically recognized cancer cells with different levels of integrin β3 expression and exhibited good serum stability. The S10yh2 aptamer also downregulates the expression of integrin heterodimers αvβ3 in cancer cells overexpressing integrin β3 and partially inhibiting cell migration. Molecular docking site analysis indicates that S10yh2 binds to 7 amino acid residues in the core region of integrin β3, supporting the unique properties of S10yh2. This aptamer is the first DNA aptamer that specifically recognizes integrin β3 and selectively binds to integrin β3 during in vitro experiments. We hope our method can provide effective tools for integrin-related tumor microenvironment studies.

## 2 Experimental Section

### 2.1 Cell Lines and Antibodies

A549 (human non-small cell lung cancer cells), U87 (human glioma cells), HeLa (human small cell lung cancer cells), and HEK-293T (human embryonic kidney cells) were obtained from the American Type Culture Collection (ATCC). All cells were cultured at 37 °C in a humidified incubator with 5% CO_2_ using Dulbecco’s modified Eagle medium (DMEM) (HyClone) supplemented with 1% penicillin–streptomycin (HyClone) and 10% fetal bovine serum (FBS) (HyClone).

The fluorescein isothiocyanate (FITC) anti-human CD61 antibody [VI-PL2] E-AB-F1166C for integrin β3, FITC anti-CD41 antibody [MEM-06] (ab21851) for integrin αllb, CD51 recombinant rabbit monoclonal antibody (SC56-07) for integrin αv, and ITGB3 rabbit monoclonal antibody (AF1444) for integrin β3 were sourced. The sc-47724, secondary antibody HRP goat anti-rabbit IgG (H + L) (As014) was used for the GAPDH antibody (0411), and m-IgGκ BP-HRP, for sc-516102, Na/K ATPase. Recombinant human integrin alpha V protein (Tagged) (ab114240), recombinant human integrin alpha-IIb (ITGA2B), and partial (639–887aa) (CSB-EP011865HU) were used. Anti-integrin alpha V beta 3 antibody [LM609]: ab190147 is a monoclonal antibody (mAb) that recognizes the human αvβ3 heterodimer. Integrin αv/β3/CD51/CD61 antibody (sc-7312, Santacruze) was also used.

### 2.2 His-ITGB3 Protein-Coated Ni-Beads Preparation

The extracellular domain of ITGB3 cDNA (79–2154 bp) was amplified via PCR from a plasmid (Addgene) and then cloned into the pcDNA 3.1/V5-His-TOPO vector (Sangon Biotech, China). The forward primer used was ATGTGTGCCTGGTGCTCTGAT, and the reverse primer was GGGACACTCTGGCTCTTCTAC. Subsequently, the His-ITGB3 recombinant protein was transiently expressed in HEK-293T cells through plasmid transfection. The entire lysate from the HEK-293T cells overexpressing His-ITGB3 was then mixed with Ni-beads (Sangon Biotech) in binding buffer (BB) (phosphate buffer with 40 mM imidazole) and allowed to incubate for 4 h at 4 °C. Afterward, the beads were washed three times with phosphate-buffered saline (PBS) to obtain His-ITGB3-coated beads, also known as ITGB3-beads.

### 2.3 Producing ITGB3 Stably Expressed A549 Cells (A549-ITGB3-OE)

The full-length cDNA of human ITGB3 was incorporated into the pcDNA 3.1/V5-His-TOPO vector (Sangon Biotech, China). The forward primer used was ATGTGTGCCTGGTGCTCTGAT, and the reverse primer was TTAAGTGCCCCGGTACGTGATATT. All plasmid sequences were validated using DNA sequencing analysis. Plasmids containing ITGB3 cDNA were introduced into A549 cells via Lipofectamine 3000 Transfection Reagent, following the manufacturer’s instructions (Thermo Fisher). After two days of transfection, cells were transferred to a selection medium containing 500 μg/mL G418 (Thermo Fisher). After three weeks of selection, cells exhibiting stable expression of ITGB3 were isolated using fluorescence-activated cell sorting (FACS) and confirmed via western blot assay.

### 2.4 SELEX Procedures

All oligonucleotides were purchased from Sangon Biotech Co., Ltd (Shanghai, China) with HPLC purification. The initial single-stranded DNA (ssDNA) library was as follows: 5′-TTCAGCACTCCACGCATAGC-40N-CCTATGCGTGCTACCGTGAA-3′. The forward primer was labeled with FAM: 5′-FAM-TTCAGCACTCCACGCATAGC-3′. The reverse primer was labeled with biotin: 5′-biotin-TTCACGGTAGCACGCATAGG-3′.

For the first round of selection, the initial ssDNA pool (5 nmol) was dissolved in 500 μL of BB (PBS with 5 mM MgCl_2_, 4.5 g/L glucose, and 100 μg/mL tRNA) and heated at 95 °C for 5 min, followed by immediate cooling on ice for at least 10 min. Subsequently, ITGB3-coated beads (about 1000 pmol of protein) were mixed with the denatured initial ssDNA pool at 4 °C for 60 min on a rotary shaker. After being washed with washing buffer (consisting of PBS with 5 mM MgCl_2_ and 4.5 g/L glucose), the ITGB3-coated beads were directly added to the PCR cocktail, which included 400 nM of FAM-labeled forward primer, 400 nM of biotin-labeled reverse primer, 100 μM of each deoxynucleotide triphosphate, and 2.5 units of KOD-Taq DNA polymerase, for subsequent amplification. The PCR protocol included an initial denaturation step at 95 °C for 3 min, followed by seven cycles of denaturation at 95 °C for 30 s, annealing at 53 °C for 30 s, and extension at 72 °C for 30 s. A final extension step was performed at 72 °C for 2 min. The biotin-labeled double-strand amplification products were captured by streptavidin-coated magnetic beads (Sangon Biotech) and denatured by 0.1 M NaOH. The supernatant containing FAM-labeled sense DNA was collected for desalting using 3 K ultrafiltration tubes (Millipore). The resulting desalted FAM-labeled sense DNA was used as a pool for the subsequent selection round. The counter-selection was performed after two rounds of selection. Before interacting with ITGB3-coated beads, the DNA pool was treated with His-tagged coated beads for 20 min to eliminate the His tag and bead-binding sequences. The supernatant with non-binding sequences was collected and incubated with ITGB3-beads. After seven rounds of selection, ITGB3-beads were replaced with ITGB3 positive A549-ITGB3-OE cells for positive selection, and His-tag beads were replaced with ITGB3 negative cell line HeLa for counter-selection. Following nine screening cycles, the resultant ssDNA library was subjected to PCR amplification using unmodified primers for next-generation sequencing (NGS) analysis by Sangon Biotech Co., Ltd (Shanghai, China).

### 2.5 Flow Cytometry Assay

Approximately 1 × 10^5^ beads or cells were incubated with FITC-labeled antibodies, Cy5-labeled aptamers, or FAM-labeled aptamers in 100 μL of BB at 4 °C for 30 min to assess the enrichment of aptamers in the DNA pools and evaluate their binding capability. The beads or cells were analyzed using flow cytometry after three washes with 1000 μL of washing buffer. The Cy5-labeled initial ssDNA pool (Cy5-Library), the FAM-labeled initial ssDNA pool (FAM-Library), and IgG were used as negative controls.

To assess aptamer affinity for cells, 1 × 10^5^ A549-ITGB3-OE cells or other cells with positive expression were exposed to different concentrations of aptamers in 100 μL of BB at 4 °C for 30 min in the absence of light. Subsequently, the cells were subjected to two washes with 1000 μL of washing buffer and then suspended in 300 μL of BB for subsequent flow cytometry analysis. The cells were analyzed using a BD FACSVerse instrument (Becton, Dickinson and Company, IN, USA), and 10,000 events were recorded. The Cy5-labeled initial ssDNA pool (Cy5-Library) and the FAM-labeled initial ssDNA pool (FAM-Library) were used as negative controls. The binding assays were conducted three times, and the binding affinity was assessed by calculating the mean fluorescence intensity after subtracting the mean fluorescence intensity of the negative control. GraphPad was used to calculate the equilibrium dissociation constants (*K*_d_) using the formula: *Y* = *B*_max_*X*/(*K*_d_ + *X*).

### 2.6 Fluorescence Imaging of Cells

Cells were incubated with Cy5-labeled aptamers at a concentration of 100 nM and FITC-labeled anti-ITGB3 antibodies at 4 °C in the dark for 30 min. After three washes with an ice-cold washing buffer, fluorescence images were obtained using laser confocal fluorescence microscopy (Leica, Germany).

### 2.7 Aptamer-Pull-Down Assay

Membrane proteins were extracted from 1 × 10^7^ A549-ITGB3-OE cells. Biotin-labeled aptamers S10yh2 or Library were allowed to interact with 20 μg of membrane proteins on ice for 30 min in 200 μL of BB. Subsequently, 200 μL of PBS buffer containing 2% formaldehyde was added to the mixture, and the incubation was continued on ice for 15 min to induce in situ crosslinking. The pull-down of aptamer-bound proteins was then performed using streptavidin-coated magnetic beads, followed by western blot assay analysis.

### 2.8 Western Blot Analysis

For western blot analysis, total cell lysate, aptamer-pulled-down proteins, or membrane proteins were used. The samples were mixed with loading buffer and heated at 100 °C for 10 min, then separated using 10% SDS-PAGE with a 5% stacking gel. The proteins were transferred onto a polyvinylidene fluoride membrane (Millipore, USA). The membrane was then blocked using 5% skimmed milk (Sangon, China) in PBS supplemented with 0.1% (v/v) Tween-20 buffer (PBST) for 1 h at room temperature. Incubation proceeded overnight at 4 °C with the appropriate primary antibody. After washing the membrane with fresh PBST at room temperature three times (5 min each), it was incubated with an HRP-conjugated secondary antibody (1:5000 dilution, Santa) at room temperature for 1 h. The membrane was washed four times with fresh PBST and detected using Super Signal West Femto Maximum Sensitivity Substrate (Thermo Fisher Scientific). The images were acquired using an Amersham Imager 600 (GE Healthcare).

### 2.9 Molecular Docking Methods

The three-dimensional structure of the aptamer was predicted using the online 3D structure prediction tool iCn3D of PDB-101, based on the DNA aptamer S10yh2 sequence. The predicted structure was then converted into a PDB file. The structure of ITGB3 was extracted from the PDB database (http://www.rcsb.org, PDB ID: 6BXJ). The active pocket for aptamer binding was predicted using CASTp software (http://sts.bioe.uic.edu/castp/index.html?201l). The docking process was performed using Rosetta8 after obtaining the 3D structures of the aptamer and its target. Initially, over 2000 rough docking conformations were produced by positioning the protein and aptamer near the binding pocket while strictly restraining the positions of all heavy atoms in the protein and aptamer systems. The initial docking conformations were then used as input for subsequent high-precision docking cycles, with protein residue side chains within the binding pocket and the entire aptamer sampling 3D conformational space. Finally, the conformation with the lowest binding energy was obtained from a maximum of 50 conformers extracted during the precise docking procedure.

### 2.10 Scratch Wound Healing Assay

A549 ITGB3-OE cells were seeded into a 6-well culture plate. After scratching the 80% confluent monolayer cells with a sterile microtip, the culture medium was immediately replaced with fresh medium to remove the dislodged cells. Different concentrations of the aptamer S10yh2 and control Lib sequences were added to the medium. Cell migration of different samples was monitored and compared to study the aptamer effect on cell migration.

### 2.11 Cell Fluorescence Microscopy Imaging

S10yh2 at a concentration of 500 nM was added to 100 μL of BB containing 20% FBS and then incubated with A549 cells (or U87, HeLa, A549-ITGB3-OE cells) on ice. The cells were washed twice with 100 μL of BB, centrifuged at 1300 rpm for 3 min, and resuspended in 100 μL of BB. The cells were then washed three times with wash buffer. The cells were observed on a thin glass slide using a 63× oil-immersion objective on a confocal microscope and imaged using a Leica laser scanning confocal microscope. The excitation source for Cy5 was a 638 nm He–Ne laser (the excitation source for fam was a 488 nm laser).

### 2.12 Single-Aptamer Secondary Labeling for Flow Cytometry

The entire process should be performed under sterile conditions with gentle handling. A549-ITGB3-OE cells that have been positively labeled with S10yh2 are collected for the first time and verified using FACS. Centrifuge the cells at 1000 rpm for 3 min, discard the supernatant, and resuspend the cells in 300 μL DPBS. Add 3 μL of DNase I (NEB, M0303S), and incubate at 37 °C for 30 min to completely degrade the aptamer. Centrifuge again at 1000 rpm for 3 min, discard the supernatant, and wash the cells three times with 1 mL PBS buffer, centrifuging at 1000 rpm for 3 min each time. Resuspend the cells in 30 μL of BB buffer and divide them into three tubes (cell only, Lib, and S10yh2), each containing 100 μL. Incubate 500 nM S10yh2 with 1 × 10^5^ A549-ITGB3-OE cells in 100 μL BB at 4 °C for 30 min. Wash the cells three times with washing buffer (WB buffer), centrifuging at 1000 rpm for 3 min each time. Analyze the cells on a BD FACSVerse instrument (Becton, Dickinson and Company, IN, USA) and record 10,000 events. Data analysis should be performed using FlowJo software (V 10.0.8r1).

### 2.13 CCK8 Cell Proliferation Experiments

The CCK-8 assay was used to measure the proliferation of A549-ITGB3-OE cells after multiple rounds of labeling. A549-ITGB3-OE cells were seeded in a 96-well plate at a density of 10,000 cells per well in 100 μL of cell culture medium. The cells were then incubated at 37 °C and 5% CO_2_ in a humidified incubator for 24 h to allow cell adhesion. The CCK-8 reagent was prepared according to the manufacturer’s instructions. The cells were washed twice with ice-cold PBS, and then 100 μL of cell culture medium containing 10% CCK-8 was added to each well. The plate was incubated at 37 °C for 30 min in the incubator. The absorbance of the mixture was measured at 450 nm using a plate reader. The experiment was repeated six times, with the cell proliferation rate calculated by normalizing the absorbance of the treated wells to that of the untreated control wells.

### 2.14 Preparation of Thiol-Modified DNA-Au Nanoparticle Complexes

Initially, thiol modification was implemented at the 5′ end of S10yh2 or Lib (Sangon Biotech, China), followed by its connection with oligoethylene glycol (OEG) spacers of varying lengths (OEG). Subsequently, the modified DNA underwent a 20 min aging period before being directly incubated with citrate-stabilized Au nanoparticles at the desired salt concentration for a duration of 10 min. The quantity of DNA loaded onto the AuNP was controlled by adjusting the ratio of DNA to gold nanoparticles to 1:4.

## 3 Results and Discussion

### 3.1 Aptamer Screening and Characterization

To find aptamers that can target integrin β3 on live cell membranes, we first planned to obtain a His-tagged extracellular domain of integrin β3 (His-ITGB3) with six histidine copies from HEK-293T cells. Western blot analysis confirmed the overexpression of ITGB3 in cell lines (Figure S1A). The recombinant His-ITGB3 protein was purified and concentrated using nickel-modified beads (Ni-Beads) for subsequent selection. Flow cytometry was used to confirm the successful purification of the ITGB3 protein on beads (Figure S1B). Meanwhile, a stable high-expression cell line of full-length ITGB3 was also established with an A549 cell line (A549-ITGB3-OE cells). Western blotting with an anti-ITGB3 antibody confirmed ITGB3 overexpression in A549-ITGB3-OE cells (Figure S2).

**Figure 1:**
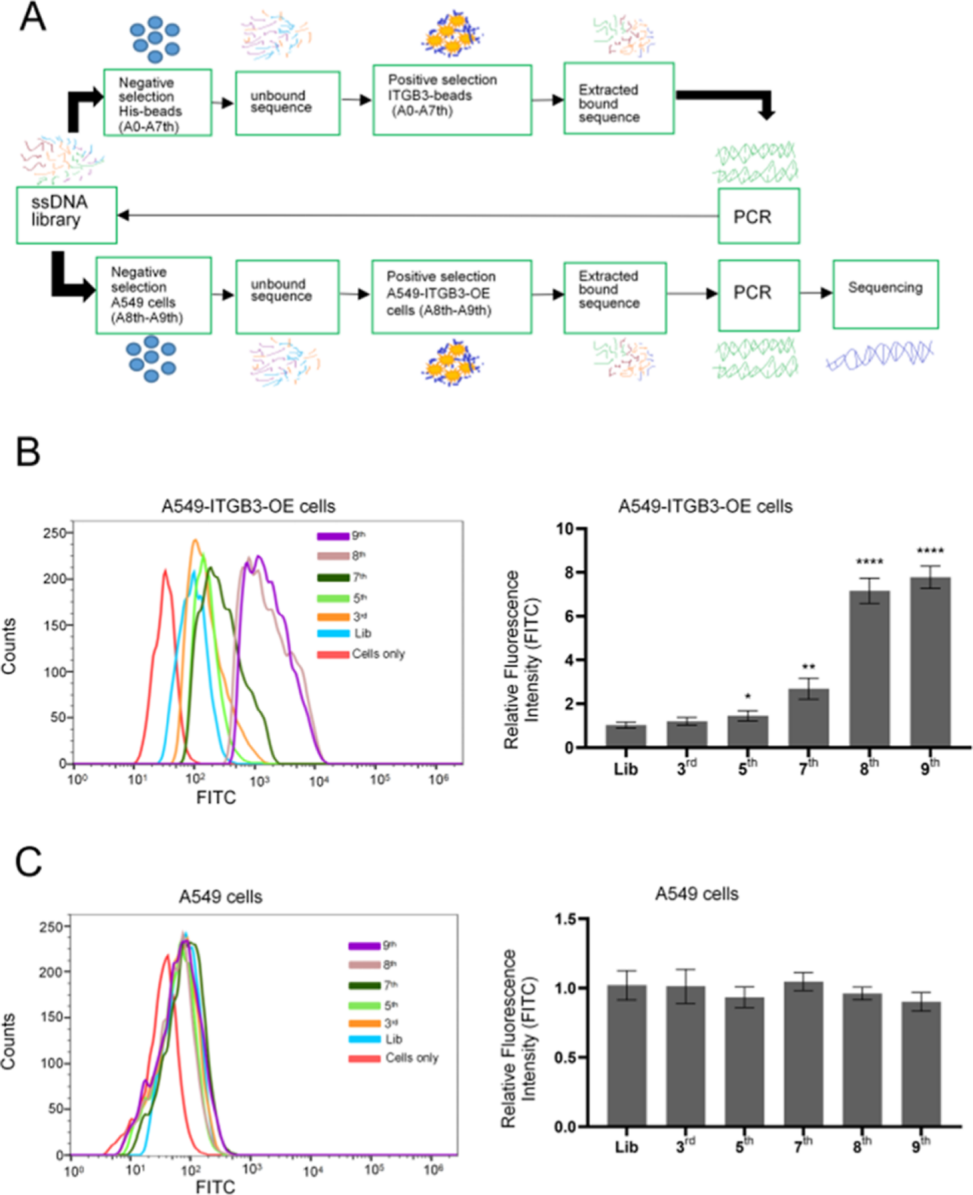
Screening process of DNA aptamers. (A) Workflow of SELEX; (B) the binding ability of enriched pools against A549-ITGB3-OE cells, compared to Lib, the significance levels obtained through a *t*-test are as follows: *P < 0.05, **P < 0.01, ***P < 0.001, and ****P < 0.0001; (C) the binding ability of enriched pools against A549 cells.

Next, a combination of protein-SELEX and cell-SELEX techniques was used to obtain aptamers that specifically recognize the ITGB3 subunit on the cell membrane. The selection process is shown in Figure 1A, where ITGB3-Beads were used as the positive selection target for the first seven rounds, and His-tagged beads (His-Beads) with six histidine copies were used as the counter-positive selection target for the first seven rounds, and His-Beads with six histidine copies were used for counter-selection to screen for high-specificity aptamers. As the number of selection rounds increased, the selection pressure was increased by reducing the library quantity and ITGB3 bead concentration. Detailed selection conditions are shown in Table S1. Flow cytometry was used to monitor the enrichment of ssDNA that binds to ITGB3 in vitro. From the 5th to the 7th round of selection, the fluorescence intensity of the ssDNA pool on ITGB3 beads gradually increased, indicating the enrichment of ssDNA that binds to ITGB3 with increased selection pressure (Figure S3). To ensure that the aptamers can also recognize ITGB3 on the surface of live cells, after seven rounds of protein-based SELEX, A549-ITGB3-OE cells with high ITGB3 expression were used as the positive selection target, and A549 cells with minimal ITGB3 expression were used for counter-selection for another three rounds. Flow cytometry analysis showed that the ssDNA pool from the 7th, 8th, and 9th rounds bound to A549-ITGB3-OE cells but not A549 cells (Figure 1B,C). These results indicate successful enrichment of aptamers targeting ITGB3 in the 7th to 9th round pools.

**Figure 2:**
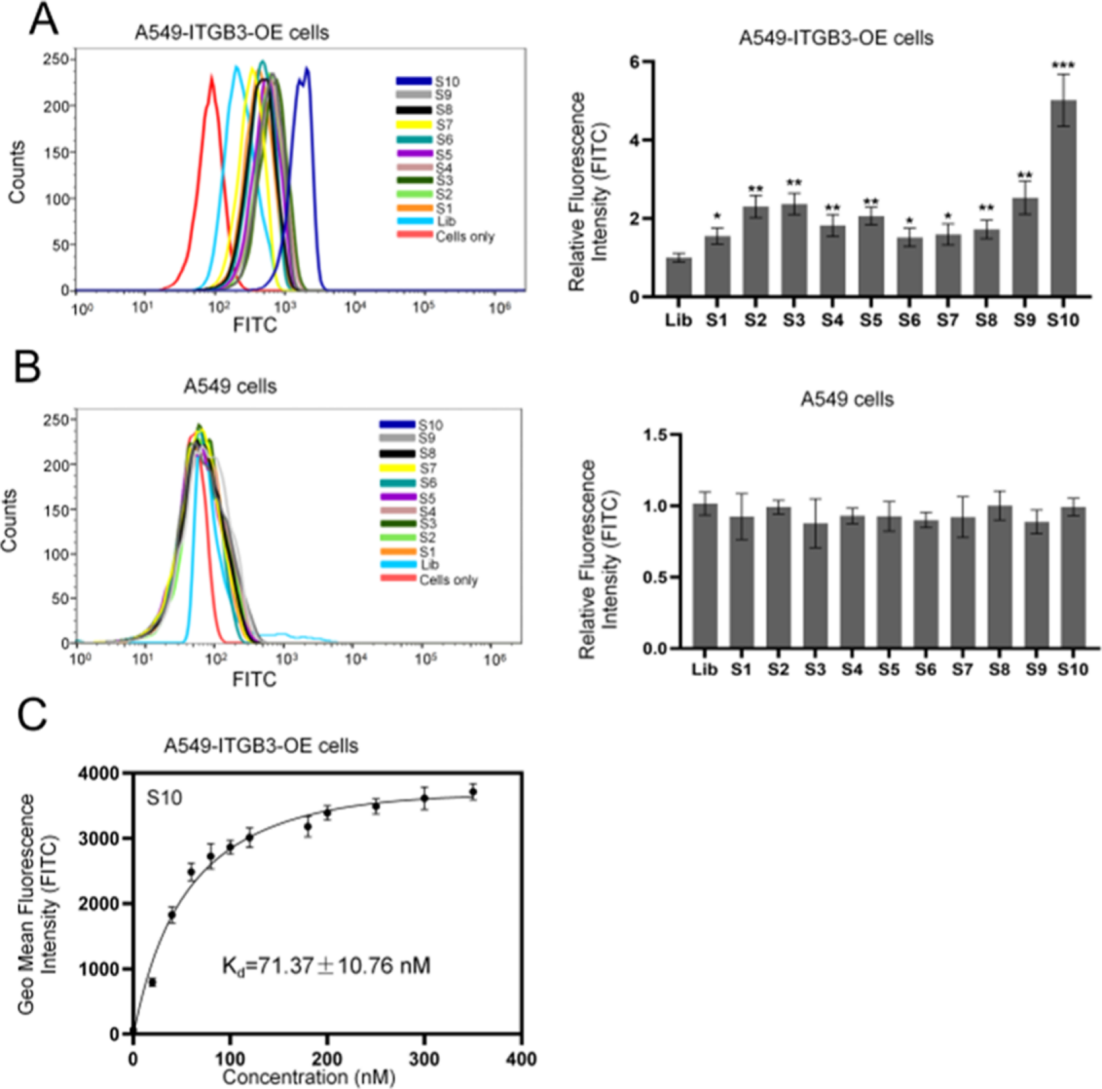
Selection of optimal DNA aptamers. (A) Binding ability of different aptamers against A549-ITGB3-OE cells, compared to Lib, the significance levels obtained through a *t*-test are as follows: *P < 0.05, **P < 0.01, and ***P < 0.001; (B) the binding ability of different aptamers against A549 cells; (C) equilibrium dissociation constant (*K*_d_) curve of aptamer S10 for A549-ITGB3-OE cells.

After being subjected to NGS, the intersection of the highly enriched sequences in the 7th, 8th, and 9th pools was compared and analyzed. According to the sequence similarity, the top 10 families of the intersection were selected for further analysis (Table S2). Flow cytometry results showed that all ten sequences had different binding abilities to A549-ITGB3-OE cells (Figure 2A) but not to A549 cells (Figure 2B). Among them, the S10 aptamer had the best binding performance to ITGB3-positive A549-ITGB3-OE cells. The *K*_d_ value of S10 for A549-ITGB3-OE cells is 71.37 ± 10.76 nM (Figures 2C and S4). These results indicate that aptamers binding to ITGB3 on cell membranes are successfully generated.

### 3.2 Truncation and Characterization of Aptamer S10

**Figure 3:**
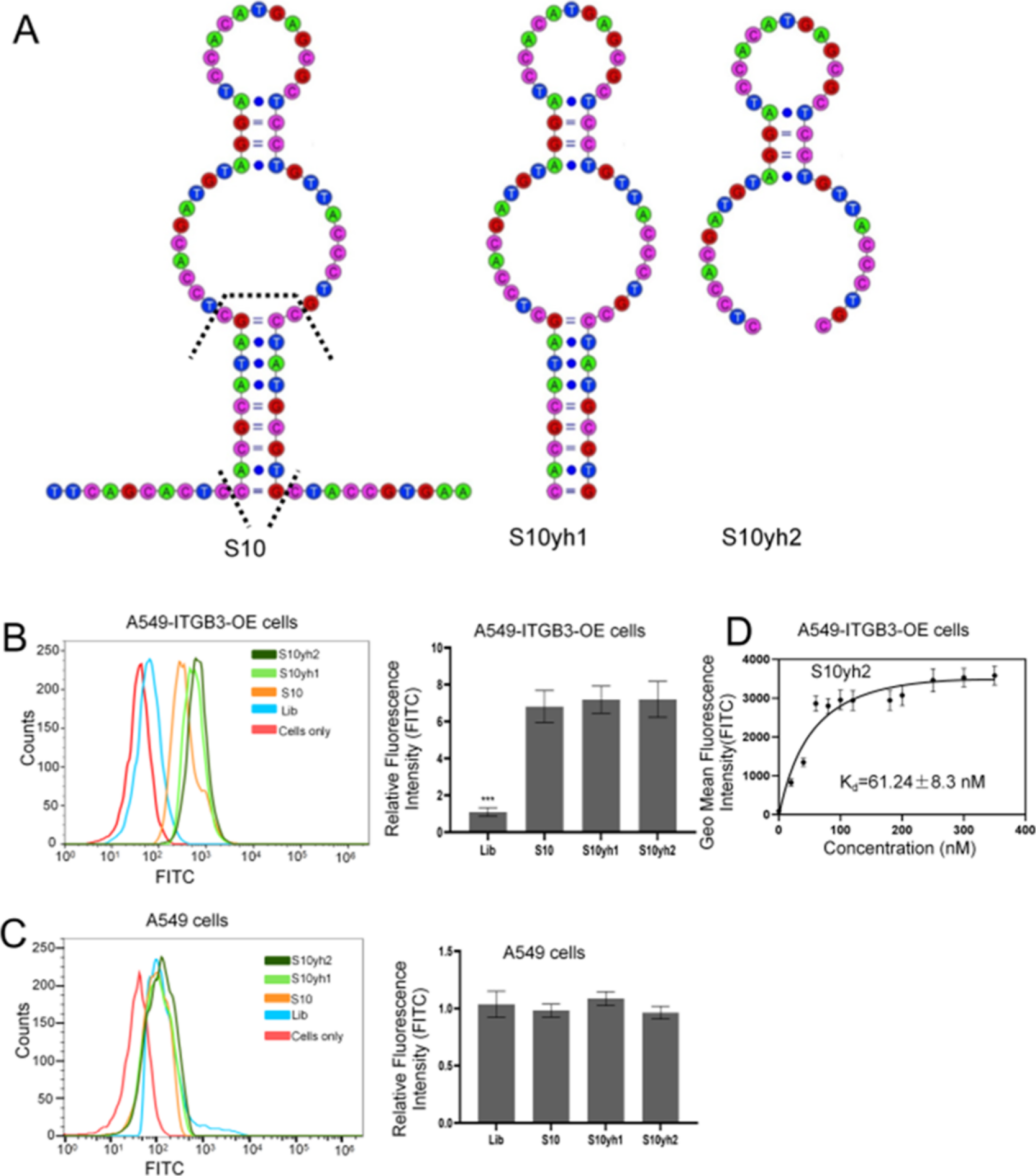
Truncation and characterization of aptamer S10. (A) Predicted secondary structure of aptamers S10, S10yh1, and S10yh2; (B) the binding ability of aptamers S10, S10yh1, and S10yh2 against A549-ITGB3-OE cells, compared to S10, the significance levels obtained through a *t*-test are as follows: ***P < 0.001; (C) the binding ability of aptamers S10, S10yh1, and S10yh2 against A549 cells; (D) the equilibrium dissociation constant (*K*_d_) curve of aptamer FAM-S10yh2 for A549-ITGB3-OE cells.

To facilitate the subsequent study of the binding sites and improve the structural stability of selected aptamers, aptamer S10 was analyzed and truncated. Specifically, the secondary structure of S10, predicted by NUPACK software, consists of a stem-loop structure with single strands at both ends (Figure 3A).^24^ Truncation was performed from both ends to generate aptamers S10yh1 and S10yh2 (Figure 3A). Flow cytometry analysis revealed that the binding affinity increased slightly with a *K*_d_ value of 61.24 ± 8.3 nM after truncating the stem and single strands at both ends (S10yh2) (Figure 3B,C) (Figures 3D and S5). S10yh1 and S10yh2 have equivalent binding abilities (Figure 3B). This result indicates that the binding site of S10 may be present in the top loop and stem zones.

**Figure 4:**
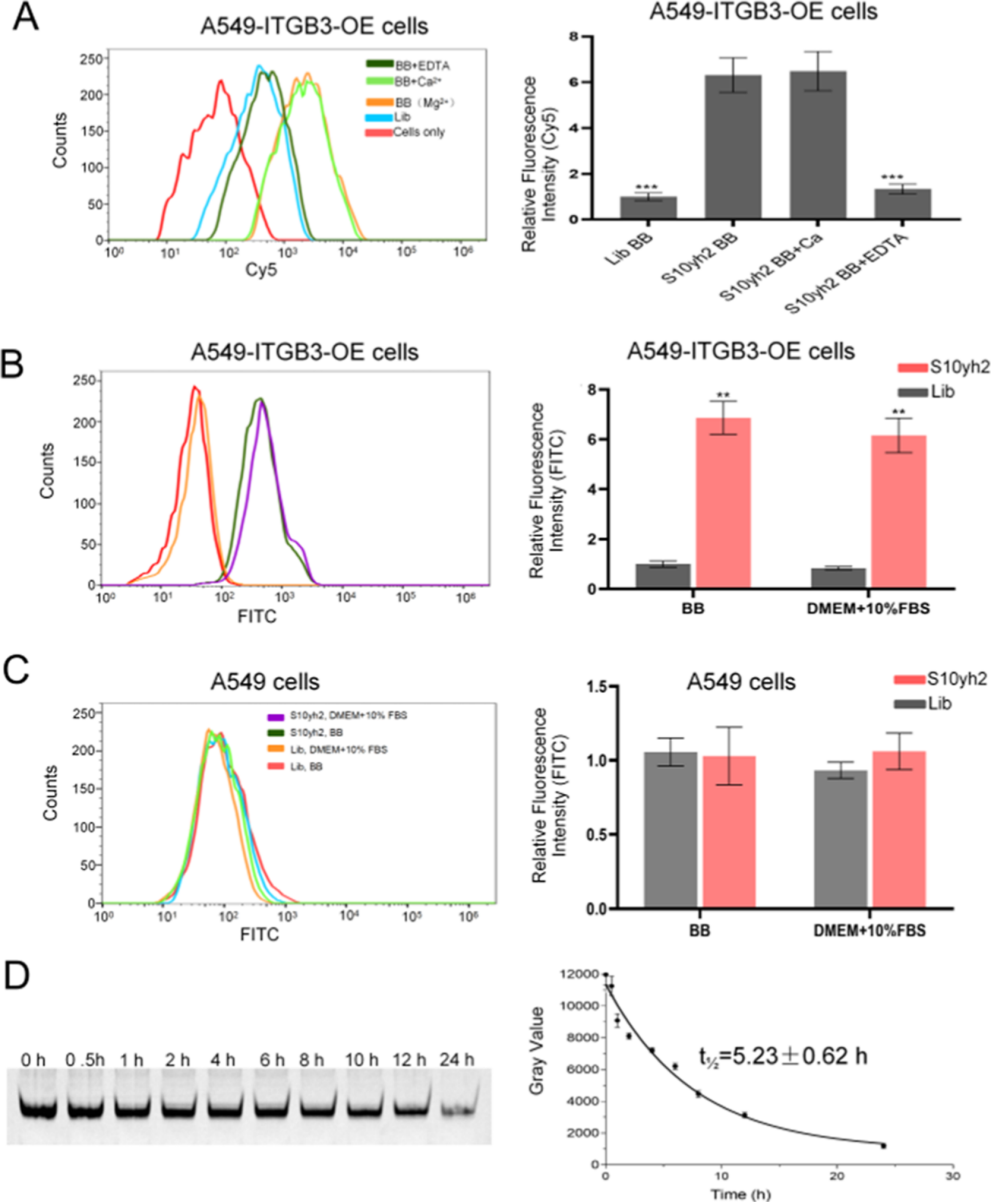
Binding conditions and stability of S10yh2. (A) Influence of divalent ions on the binding ability of S10yh2 on A549-ITGB3-OE cells. (B) Influence of serum on the binding ability of S10yh2 on A549-ITGB3-OE cells, compared to Lib, the significance levels obtained through a *t*-test are as follows: **P < 0.01 and ***P < 0.001; (C) the influence of serum on the binding ability of S10yh2 on A549 cells; (D) the stability of S10yh2 in DMEM + 10% FBS.

Since aptamers are selected in a BB (containing 5 mM MgCl_2_) at 4 °C, the influence of divalent ions, temperature, and serum on the binding ability of S10yh2 was investigated. Flow cytometry results show that adding Ca^2+^ (5 mM Ca^2+^) to the BB containing Mg^2+^ did not affect the binding ability of S10yh2. However, adding EDTA, which chelates Mg^2+^ in the BB, weakened the binding ability of the aptamer to the target protein (Figure 4A), indicating that the target binding ability of S10yh2 requires the presence of divalent ions. Moreover, the fluorescence intensity of cells incubated with S10yh2 had no significant change in BB or on DMEM cell culture medium containing 10% FBS at 4 °C (Figure 4B,C). Agarose gel electrophoresis analysis showed that unmodified S10yh2 was quite stable in DMEM cell culture medium containing 10% FBS, with a half-life (*t*_1/2_) of 5.23 ± 0.62 h (Figure 4D). These results indicated that S10yh2 holds great potential for biomedical applications.

### 3.3 Target Verification of Aptamer S10yh2

**Figure 5:**
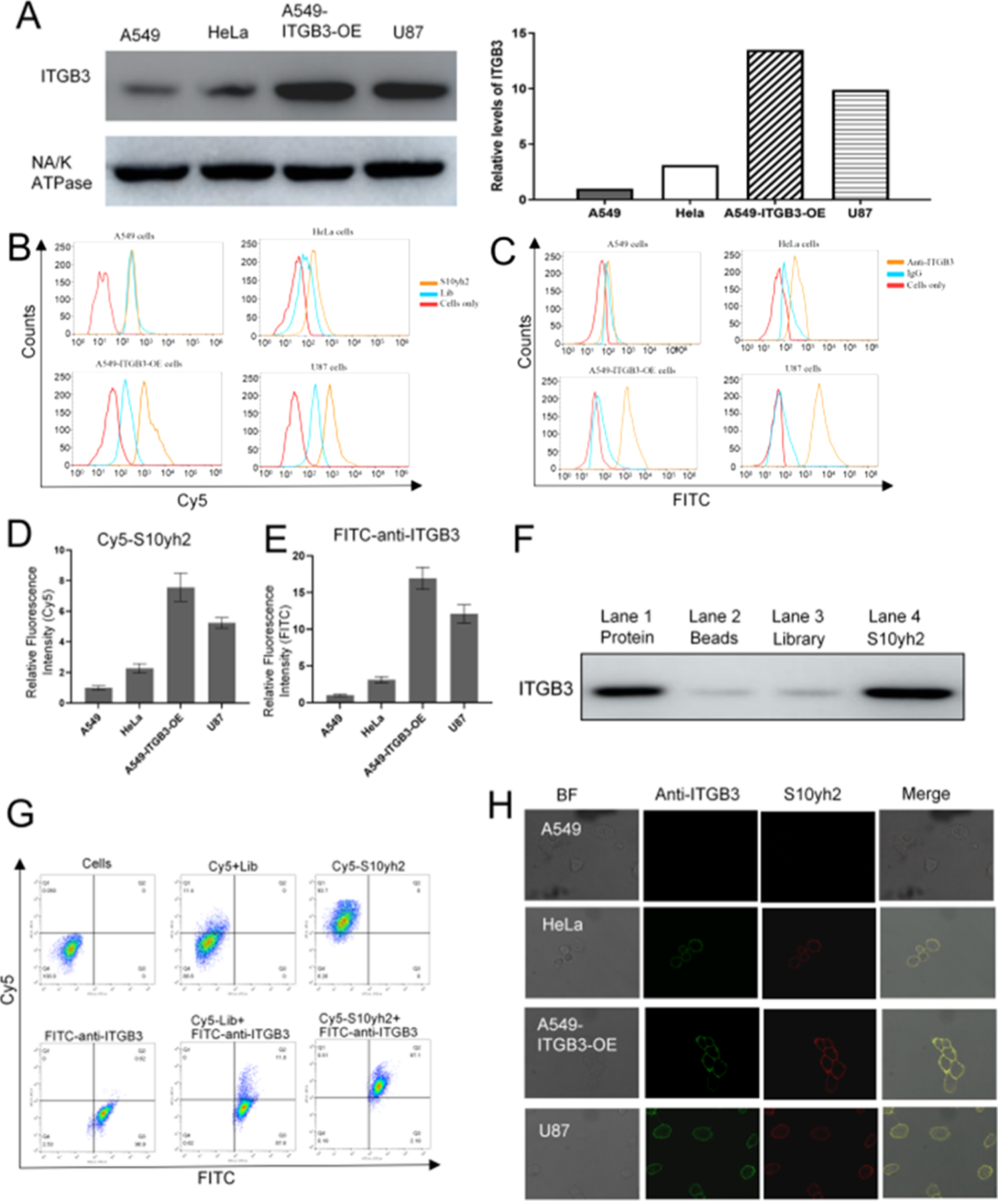
S10yh2-specific recognition of the expression levels of ITGB3 on the membrane surface of different cancer cells. (A) Western blot analysis was performed on membrane proteins extracted from the specified cell lines using anti-ITGB3 or anti-NA/K ATPase antibodies. The relative levels of ITGB3 expression compared to NA/K ATPase expression were quantified; (B) the interaction between S10yh2 and the designated cell lines was assessed via flow cytometry, employing an aptamer concentration of 500 nM and an incubation time of 30 min; (C) the cell membrane expression of ITGB3 in the specified cell lines was examined through flow cytometry using an anti-ITGB3 antibody; (D) relative fluorescent intensities of Cy5-S10yh2 were shown; (E) relative fluorescent intensities of FITC-anti-ITGB3 were shown; (F) western blot analysis of S10yh2 pulled-down protein with the ITGB3 antibody was performed. Membrane proteins extracted from A549-ITGB3-OE cells (lane 1) were incubated with beads (lane 2), beads coupled with a library (lane 3), and beads coupled with S10yh2 (lane 4); (G) U87 cells were stained with Cy5-labeled library, Cy5-labeled S10yh2, and/or FITC-labeled anti-ITGB3 antibody, and the fluorescence signals from Cy5 and FITC were subsequently assessed via flow cytometry; (H) confocal imaging of cells with FITC-labeled ITGB3 antibody and Cy5-labeled S10yh2 was performed.

Western blot analysis was undertaken to identify the ITGB3 expression levels in four cancer cell lines (Figure 5A). A Cy5-labeled S10yh2 was used to detect the expression of ITGB3 on the cell membrane of U87, HeLa, A549, and A549-ITGB3-OE cells. Flow cytometry analysis shows that S10yh2 could bind to ITGB3-positive cancer cell lines (A549-ITGB3-OE, U87) and ITGB3-medium expressing cervical cancer cell line (HeLa) but not to ITGB3-negative expressing cancer cell line (A549) (Figure 5B,D). Flow cytometry results show that the fluorescence intensity of S10yh2 in different cell lines correlated closely to FITC-anti-ITGB3 antibody labeling and western blot results (Figure 5C,E).

Furthermore, the pull-down assay confirmed the specific binding of S10yh2 to the integrin β3 subunit. The cell lyses from A549-ITGB3-OE cells were incubated with agarose beads coupled with S10yh2 or library (Lib). The pulled-down proteins were detected using western blots with a specific anti-ITGB3 antibody. As shown in Figure 5F, S10yh2-coated beads effectively pulled down ITGB3, while library-coated beads did not show significant bands (Figure 5F, lane 2 and lane 3), indicating the specific interaction between S10yh2 and ITGB3. These findings ultimately confirm the binding of S10yh2 to the ITGB3 protein on the cell membrane.

In order to investigate the potential co-binding of S10yh2 and the ITGB3 antibody, U87 cells were simultaneously exposed to Cy5-labeled S10yh2 or FITC-labeled anti-ITGB3 antibody. Flow cytometry analysis demonstrated a direct correlation between the fluorescence signals observed in the Cy5 and FITC channels within U87 cells (Figure 5G). Additionally, the distribution of FITC-anti-ITGB3 antibody and S10yh2 on tumor cells was also examined. Cells were co-incubated with FITC-anti-ITGB3 antibody and Cy5-labeled S10yh2, and confocal imaging demonstrated a strong overlap in fluorescence signals between FITC-labeled anti-ITGB3 antibody and Cy5-labeled S10yh2 on the surfaces of HeLa cells, A549-ITGB3-OE cells, and U87 cells (Figure 5H). Conversely, no significant fluorescence signal was detected on A549 cells. These findings suggest that S10yh2 and the antibody are capable of binding to distinct sites on the extracellular domain of ITGB3 simultaneously.

### 3.4 Characterization of S10yh2 and ITGB3 Binding with Computational Modeling Analysis

**Figure 6:**
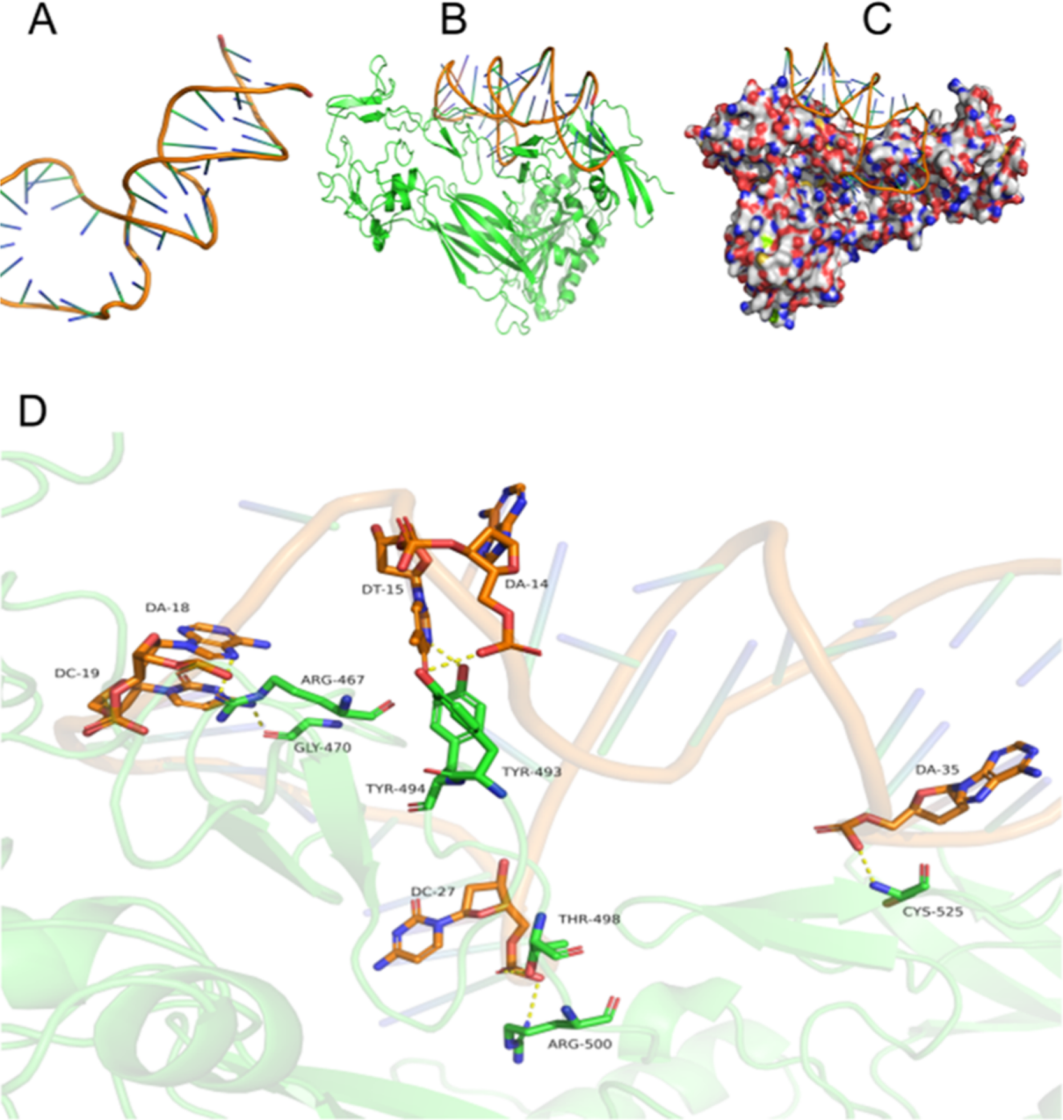
Binding mode prediction between ITGB3 and S10yh2. (A) Structure of S10yh2; (B) close view of ITGB3 and S10yh2 interaction; (C) conformation superposition of crystal ITGB3 and S10yh2; (D) binding pose between ITGB3 and S10yh2 obtained by precise docking.

Molecular details of the binding site between S10yh2 and ITGB3 are ascertained via computational modeling analysis. In blind docking, 2000 conformations were extracted and sorted based on their docking energy. Functional sites for the interaction of the target protein ITGB3 and the selected aptamer S10yh2 were calculated using the 200 conformations with the lowest binding energies (Figure 6A). Multiple conformational superposition analyses reveal that the binding sites of ITGB3 and S10yh2 are highly localized (Figure 6B,C). Fourteen potential binding residues with relatively high ratios (*R* > 0.4) were initially identified as the binding residues of S10yh2 for subsequent precise docking. Detailed analysis uncovered that S10yh2 could strongly associate with seven residues, including CYS525, THR498, ARG500, TYR493, TYR494, ARG467, and GLY-470. The complex structure between ITGB3 and S10yh2 was extracted for further analysis using precise docking methods. Conformational superposition analyses reveal that the binding sites of ITGB3 and S10yh2 are highly localized (Figure 6B,C). Fourteen potential binding residues with relatively high ratios (*R* > 0.4) were initially identified as the binding residues of S10yh2 for subsequent precise docking. Detailed analysis uncovered that S10yh2 could strongly bind to seven residues, including CYS525, THR498, ARG500, TYR493, TYR494, ARG467, and GLY-470. The complex structure between ITGB3 and S10yh2 was extracted for further analysis using precise docking methods.

The findings from the precise docking analysis prompted a 10 ns molecular dynamics simulation to investigate the dynamic behavior of the ITGB3-S10yh2 complex. Refined binding models were extracted from the trajectory, revealing that the binding interface predominantly comprises seven specific amino acids in ITGB3 that formed crucial interactions with S10yh2 and were identified as critical residues. The stability of the complex is maintained by non-covalent electrostatic interactions, including polar interactions that form a robust hydrogen bonding network at the interface between ITGB3 and S10yh2. Each region is shown in Figure 6D. Six nucleic acid bases and seven ITGB3 residues jointly form a hydrogen bond network, (A35 + CYS525, C27 + THR498, C27 + ARG500, A14 + TYR493, T15 + TYR494, A18 + ARG467, C19 + GLY-470). These data confirmed that the top loop and stem zones are keys for binding and are consistent with the data of similar binding affinity between S10 and truncated S10yh2.

### 3.5 S10yh2 Can Dynamically Respond to Changes in the Amount of Integrin β3 on the Cell Membrane

**Figure 7:**
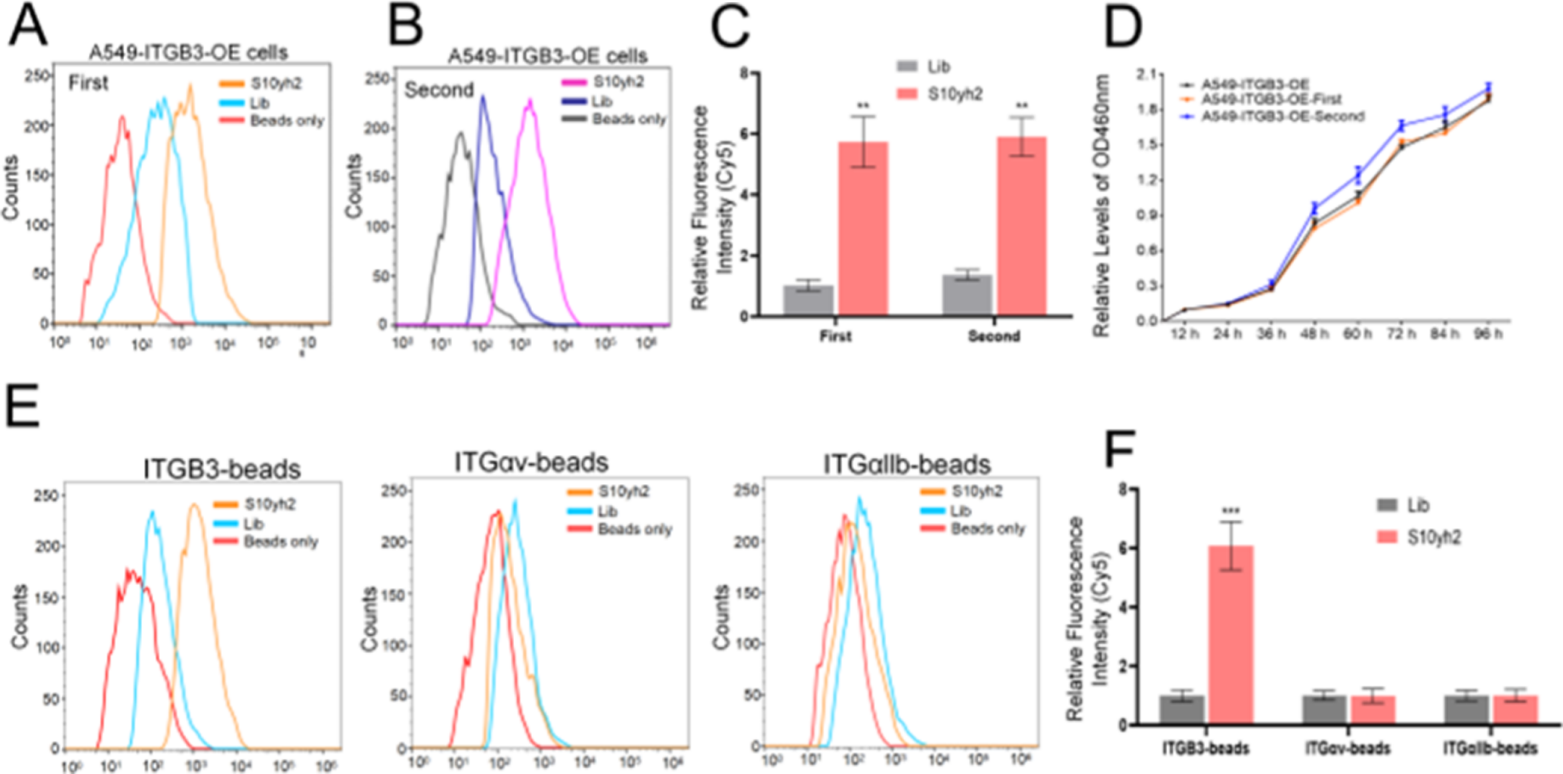
S10yh2 can dynamically respond to changes in the amount of integrin β3 on the cell membrane. (A–C) S10yh2 can repeatedly label living cells, compared to Lib, the significance levels obtained through a *t*-test are as follows: **P < 0.01 and ***P < 0.001; (D) S10yh2’s effect on cell proliferation after multiple repeated labeling treatments; (E,F) the S10yh2’s specificity for monomeric proteins compared to antibodies.

Due to the use of 4% paraformaldehyde for fixing cells in ITGB3 antibody labeling, which is unsuitable for repetitive labeling of live cells, whether S10yh2 can be used for repetitive labeling of live cells was investigated. Cells labeled with S10yh2 were first evaluated for labeling efficiency using flow cytometry (Figure 7A). Positively labeled cells were collected and treated with DNase I at room temperature for 30 min to digest DNA chains in the digestion solution (Figure 7B, cells only). Subsequently, cells were labeled with S10yh2 for the second time (Figure 7B, S10yh2). Flow cytometry results show that cells labeled with S10yh2 for the first time can be labeled with S10yh2 again following DNase I digestion. The labeling efficiency is comparable between the two labeling rounds (Figure 7C). In addition, no significant change in cell proliferation ability between cells labeled for the first and second time is identified compared to untreated cells (Figure 7D). Finally, the specificity of S10yh2 and ITGB3 antibodies for the monomeric integrin subunits is investigated. Flow cytometry results show that S10yh2 (Figure 7E) and ITGB3 antibody (Figure S6) both fail to recognize integrin αv and αllb subunits, further confirming the specific recognition ability of S10yh2 for the β3 subunit on the cell membrane surface. Concurrently, Table S4 provides a comparative examination of the biological characteristics of the S10yh2 aptamer in comparison to previously documented alphav beta3 aptamers.^19,22^ A preceding investigation has documented that RNA aptamers selectively identify the αV or β3 subunits of integrin αVβ3. These aptamers demonstrate affinities in the low nanomolar range toward their targets while displaying minimal cross-reactivity toward other closely related integrin homologues.^19^ Additionally, our research reveals that the S10yh2 aptamer demonstrates a significant affinity in the low nanomolar range toward its specific targets while exhibiting negligible cross-reactivity toward closely related integrin homologues.

### 3.6 Inhibition of the Migration of Cancer Cells with S10yh2

**Figure 8:**
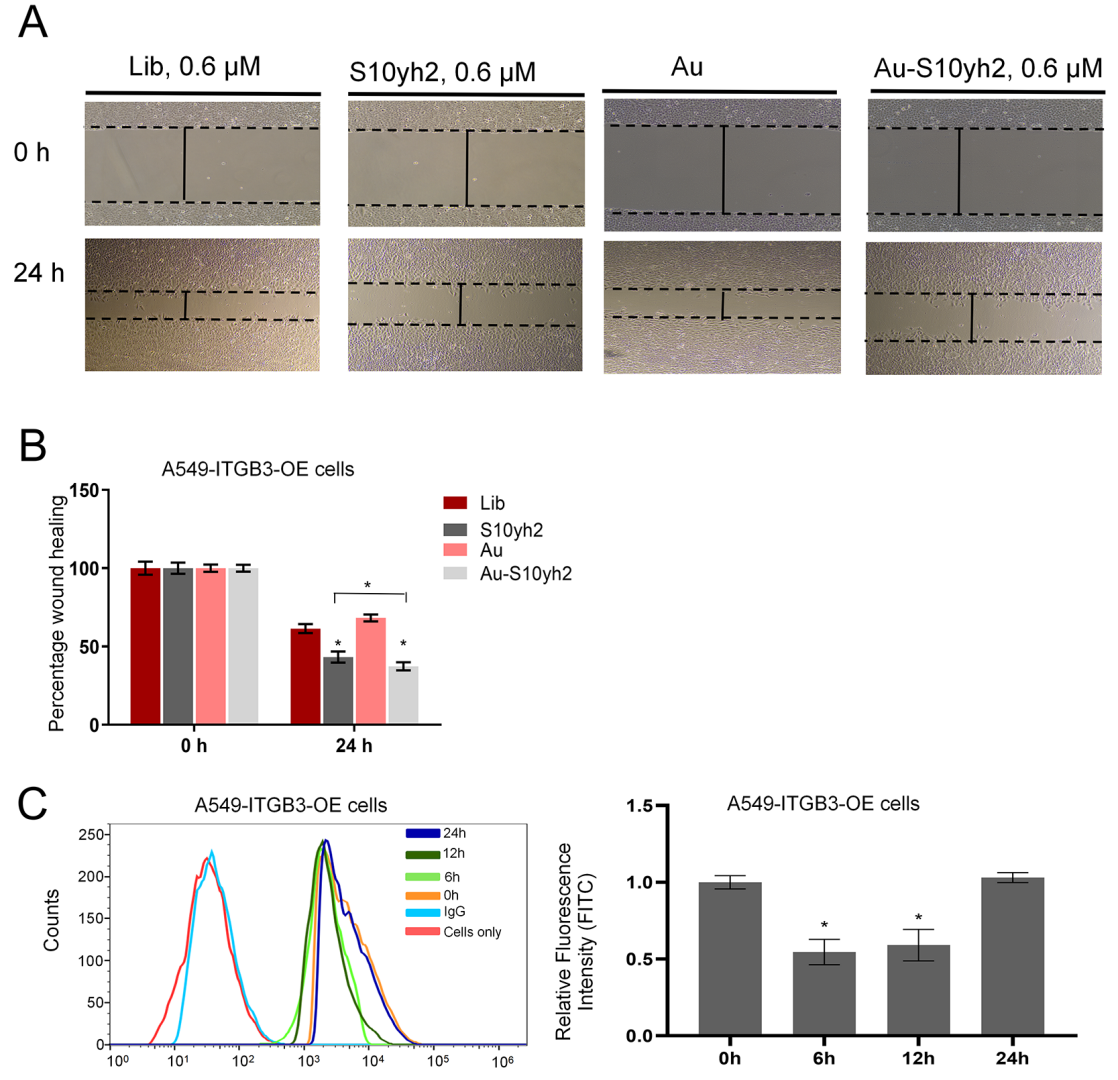
S10yh2 partially inhibits tumor cell migration. (A) Scratch wound healing assay of A549-ITGB3-OE cells with 0.6 μM S10yh2, Lib, Au-S10yh2, or Au treatment. (B) Percentage wound healing assay. Compared to Lib or Au, the significance levels obtained through *t*-tests are as follows: *P < 0.05; (C) flow cytometry analysis of the protein dimer integrin αvβ3 in A549-ITGB3-OE cells with 0.6 μM S10yh2 treatment. Compared to 0 h, the significance levels obtained through *t*-tests are as follows: *P < 0.05.

Integrin αvβ3 is highly expressed in tumor cells, which promotes tumor angiogenesis and leads to multiple site metastasis, including bone metastasis.^25^ Blocking or inhibiting tumor integrin αvβ3 has more vigorous antiangiogenic and antitumor activity.^26^ Considering the specific binding of S10yh2 to ITGB3 and the critical role of integrin αvβ3 in tumor metastasis, S10yh2 migration inhibition was tested using a scratch wound healing assay involving A549-ITGB3-OE cells. Figure 8A shows that the gap closure rate of the S10yh2-treated cells is significantly slower than the Library sequence-treated cells after 24 h. In the scratch healing assay, S10yh2 was also exposed to modification with Au nanoparticles (Figure S7). Figure 8A,B demonstrates that the Au-S10yh2 group displayed more significant inhibitory effects compared to the S10yh2 only, library, and Au only groups. Nevertheless, there is still potential for further improvement in the inhibitory efficacy of the aptamer. The enhancement of the inhibitory potency of aptamer S10yh2 can be further explored by integrating degradation technology alongside aptamer S10yh2, with the objective of attaining specific degradation of integrin β3. Further flow cytometry illustrates that 0.6 μM S10yh2 treatment inhibited the formation of αvβ3 heterodimers (Figure 8C). The inhibitory effect continued to increase within 12 h and gradually weakened between 12 and 24 h (Figure 8C).

Since the half-life of S10yh2 in DMEM cell culture medium with 10% FBS is 5.23 ± 0.62 h (Figure 4C), the degradation potentially causes the gradually decreasing inhibitory effect. Bennett reported that the binding hot spots of the β3 subunit to αν include Lys532 to Gly690,^27^ and partially coincide with the S10yh2 binding residues. These data infer that S10yh2 may induce steric hindrance at the critical binding site of integrin β3, thus inhibiting heterodimer formation, indicating that S10yh2 binding to ITGB3 interferes with heterodimer formation between αv integrin and ITGB3, thus inhibiting the tumor cell migration. Due to its substantial binding and migration inhibition abilities against ITGB3-expressed cancer cells in complex environments, S10yh2 holds excellent potential for clinical application.

The research findings indicate that, unlike the ITGB3 antibody, S10yh2 labeling does not require cell fixation and does not significantly impact cell viability during the labeling process. Therefore, S10yh2 is suitable for repetitive labeling of live cells to dynamically monitor changes in integrin β3 expression on the cell membrane. Its labeling efficiency remains comparable even after DNase I digestion. Furthermore, the research results also demonstrate that the results remain comparable even after DNase I digestion. Furthermore, the research results also demonstrate that S10yh2 specifically recognizes the β3 subunit on the cell membrane surface without recognizing other integrin subunits such as αv or αllb, indicating a high degree of selectivity in the specific recognition of the β3 subunit by S10yh2. These findings suggest that S10yh2 can label live cells repetitively, specifically recognizing the β3 subunit on the cell membrane surface. There is no significant impact on cell viability, making it an important tool for studying dynamic changes in integrin β3 expression on the cell membrane and other related research applications.

## 4 Conclusions

ITGB3, as an extensively studied member of the integrin family, plays critical and diverse roles in the progression of malignant tumors and the reprograming of the tumor microenvironment. These roles include metabolic reprograming, endothelial-to-mesenchymal transition (End–MT), epithelial-to-mesenchymal transition (EMT), acquisition of drug resistance, regulation of stemness, re-education of the stromal and immune microenvironment, and pro-angiogenesis. In scenarios such as TGF-β induced EMT and tumor-initiating cells, ITGB3 is upregulated, leading to increased migration, invasion, maintenance of stemness, and consequent resistance to targeted therapies. Currently, anti-ITGB3 drugs, including cilengitide, vitaxin, and MK0429, are in clinical trials.^1^ However, as peptidic drugs, cilengitide and vitaxin are challenging to modify chemically and have strong immunogenicity.^28,29^ The targeting specificity of the small molecule antagonist drug MK-0429 is poor.^30^

This study used a combination of protein-SELEX and cell-SELEX techniques to design and produce a high-specificity and high-affinity ssDNA aptamer, S10yh2, for specific recognition of the ITGB3 subunit on the cell membrane through rigorous screening and careful truncation. The *K*_d_ value of S10yh2 against ITGB3 highly expressed A549-ITGB3-OE cells is 61.24 ± 8.3 nM. S10yh2 retained activity in the DMEM cell culture medium with 10% FBS, with a half-life-time of 5.23 ± 0.62 h. Furthermore, S10yh2 treatment inhibited the formation of the integrin αvβ3 heterodimer and the migration of A549-ITGB3-OE cells. Furthermore, S10yh2 can repeatedly label live cells, specifically recognizes the β3 subunit on the cell membrane surface, and does not significantly affect cell viability, making it a valuable tool for studying the dynamic changes in integrin β3 expression on the cell membrane and other related research applications. All the data from this study suggest that the aptamer S10yh2 holds great potential as a clinical tool for probing and treating ITGB3 abnormally expressed tumors.

## Supporting Information Available

Detailed SELE conditions; major groups of sequences in the 9^th^ library; optimization and mutated sequence of aptamer S10; comparative analysis of the biological attributes of the S10yh2 aptamer in relation to previously reported αvβ3 aptamers; western blot analysis of ITGB3 expression in different cell lines; monitoring the enrichment of aptamers during SELEX by FAC; monitoring the binding curves of FAM-S10 aptamer to A549-ITGB3-OE cells by FACS; flow cytometry assay of beads costained by FITC-anti-ITGα; and electron microscopy image of Au (PDF)
