# Supplementary material for: Screening a DNA Aptamer Specifically Targeting Integrin β3 and Partially Inhibiting Tumor Cell Migration
Source: Anal Chem. 2023 Aug 9;95(33):12406–18. doi: 10.1021/acs.analchem.3c01995 (PMC10448441; doi:10.1021/acs.analchem.3c01995)
Supplement: Supplementary file 1 — ac3c01995_si_001.pdf [file ac3c01995_si_001.pdf]

# Supporting information

# Screening a DNA Aptamer Specifically Targeting Integrin β3 and Partially Inhibiting Tumor Cell Migration

Xiaoyan Teng,‡[a] Yu Wang,‡[b] Liuxia You,‡[c] Lirong Wei,[a] Chao Zhang,*[b] and Yuzhen Du*[a]

AUTHOR ADDRESS

[a]Department of Laboratory Medicine, Shanghai Jiao Tong University Affiliated Sixth People's Hospital, Shanghai 200233, China.

[b]State Key Laboratory of Oncogenes and Related Genes, Shanghai Cancer Institute, Department of Oncology, Institute of Molecular Medicine, Renji Hospital, School of Medicine, Shanghai Jiao Tong University, Shanghai, 200127, China.

[c]Department of Clinical Laboratory, The Second Affiliated Hospital of Fujian Medical University, Quanzhou, Fujian, 362000, China.

‡Xiaoyan Teng, Yu Wang and Liuxia You have contributed equally to this work.

Table S1. Detailed SELEX conditions.

| Rounds | Library (pmol) | Selection target | Counter selection target |
|---|---|---|---|
| 1 | 5 OD | ITGB3-beads | - |
| 2 | 200 | ITGB3-beads | His Tag-beads |
| 3 | 200 | ITGB3-beads | His Tag-beads |
| 4 | 150 | ITGB3-beads | His Tag-beads |
| 5 | 125 | ITGB3-beads | His Tag-beads |
| 6 | 100 | ITGB3-beads | His Tag-beads |
| 7 | 100 | ITGB3-beads | His Tag-beads |
| 8 | 100 | A549-ITGB3 cells (60 mm dish) | A549 cells (60 mm dish) |
| 9 | 100 | A549-ITGB3 cells (60 mm dish) | A549 cells (60 mm dish) |

Table S2. Major groups of sequences in the 9th library.

| Name | Sequence | Percentage |
|---|---|---|
| Library | TTCAGCACTCCACGCNNNNNNNNNNNNNNNNNNNNNNNNN NNNNNNNNNNNNNNNCCTATGCGTGCTACCGTGAA | - |
| S1 | TTCAGCACTCCACGCATAGCTGGCTATTCCGAGATTCCTTGC GCGAACTTCGTTGCTACACCTATGCGTGCTACCGTGAA | 20 % |
| S2 | TTCAGCACTCCACGCATAGCACATGTAGTGTGTGCGCCACA ATTTGGGGCAACCAACTGACCTATGCGTGCTACCGTGAA | 13 % |
| S3 | TTCAGCACTCCACGCATAGCATACCCTGCCGCAAGCAGCTA ATATTGATTAGTGACCGCCCCTATGCGTGCTACCGTGAA | 12 % |
| S4 | TTCAGCACTCCACGCATAGCAGCCGAAGCGACAAGCTTGTG TAGAAGTTGGAAGCGTCCGCCTATGCGTGCTACCGTGAA | 9 % |
| S5 | TTCAGCACTCCACGCATAGCTTCTGACTGAATCGCTCGGTG TCGAAACAGGTGAGCCTTACCTATGCGTGCTACCGTGAA | 8 % |
| S6 | TTCAGCACTCCACGCATAGCTGTGTTCACAAGATAAAGGGC TCCTAGGCTATGTGCGTCTCCTATGCGTGCTACCGTGAA | 7 % |
| S7 | TTCAGCACTCCACGCATAGCGAAACGGTGCCATAAGATCAA GCCACATGACATGACCTTCCCTATGCGTGCTACCGTGAA | 6 % |
| S8 | TTCAGCACTCCACGCATAGCGAGACACGCAGTGTAGATGGT CAGGGCGTAGTTGAGCTTGCCTATGCGTGCTACCGTGAA | 6 % |
| S9 | TTCAGCACTCCACGCATAGCTGACTGCTCCTTAGGTGTGAC CTAAAGTGTTCTGTTTTCGCCTATGCGTGCTACCGTGAA | 4 % |
| S10 | TTCAGCACTCCACGCATAGCTCCACGATGTAGGATCCACAT GAGCGCTCCTGTTACCCTGCCTATGCGTGCTACCGTGAA | 3 % |

Table S3. Optimization and mutated sequence of aptamer S10.

| Name | Sequence | Length |
|---|---|---|
| S10yh1 | TTCACGCATAGCTCCACGATGTAGGATCCACATGA GCGCTCCTGTTACCCTGCCTATGCGTGAA | 64 nt |
| S10yh2 | TCCACGATGTAGGATCCACATGAGCGCTCCTGTTA CCCTG | 40 nt |

Table S4. A comparative analysis of the biological attributes of the S10yh2 aptamer in relation to previously reported αvβ3 aptamers.

| Aptamer | Targeting | Biological Properties | Reference |
|---|---|---|---|
| S10yh2 (ssDNA) | Integrin β3 | Inhibition of cell migration | This study |
| αv (RNA) | Integrin β3 | High affinities and minimal cross-reactivity. | Reference 19 |
| β3 (RNA) | Integrin β3 | High affinities and minimal cross-reactivity. | Reference 19 |
| αvβ3 (ssDNA) | Integrin αvβ3 | Attenuates Vascular Smooth Muscle Cell Proliferation and Migration. | Reference 22 |

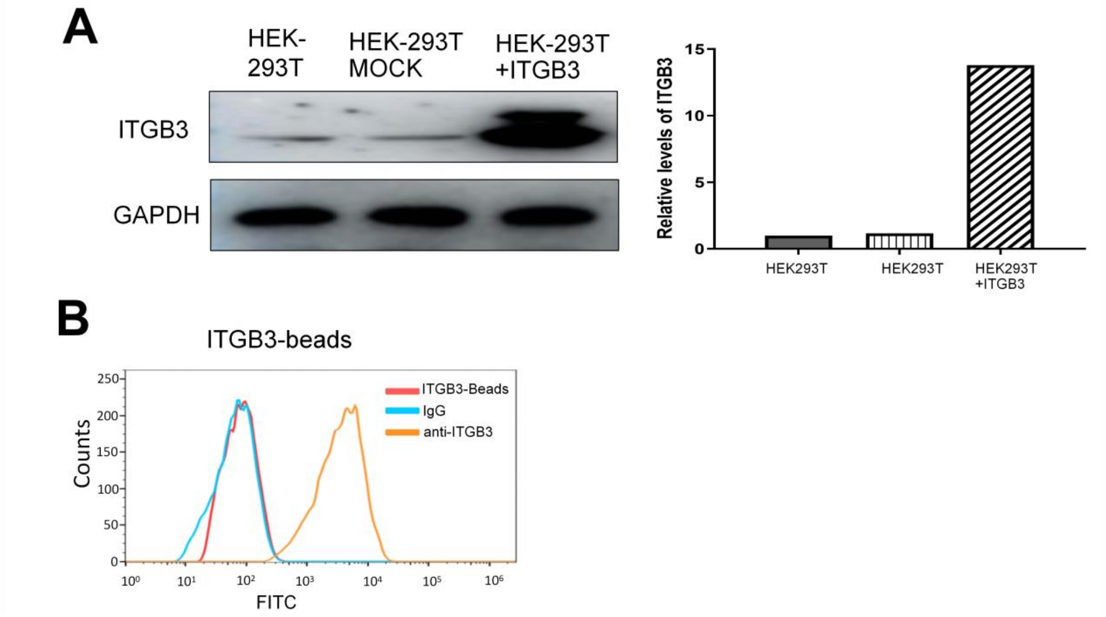


**Figure S1.** (A) Western blot analysis of ITGB3 expression in different cell lines; (B) Flow cytometry assay of beads were costained by FITC-anti-ITGB3.

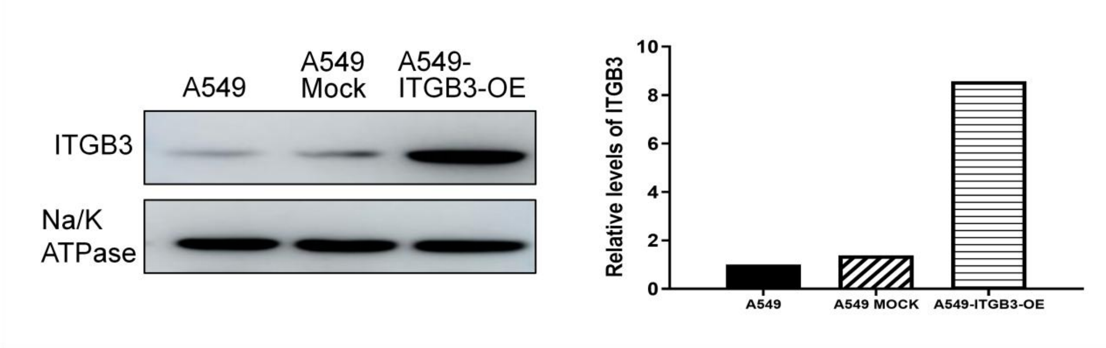


**Figure S2.** Western blot analysis of ITGB3 expression in different cell lines.

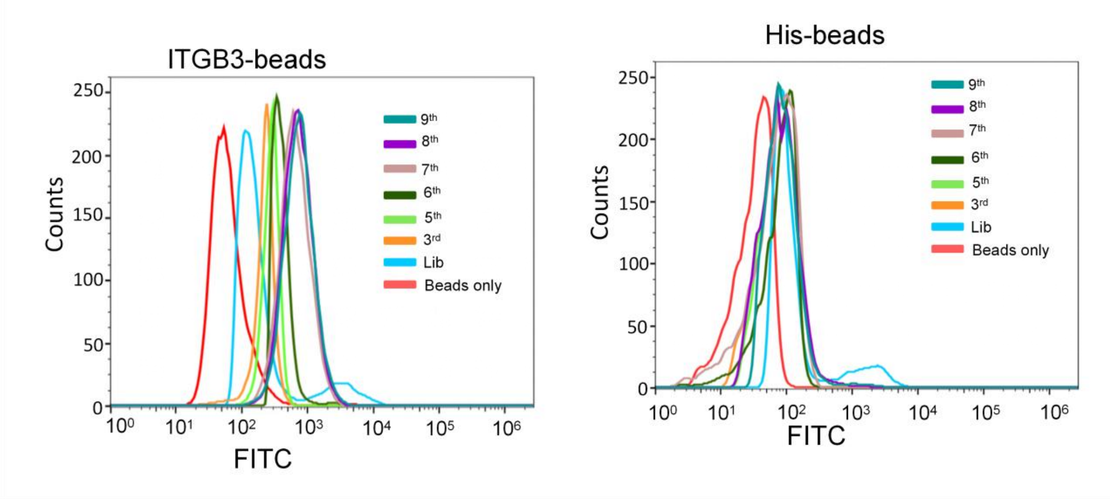


**Figure S3.** Monitoring the enrichment of aptamers during SELEX by FACS.

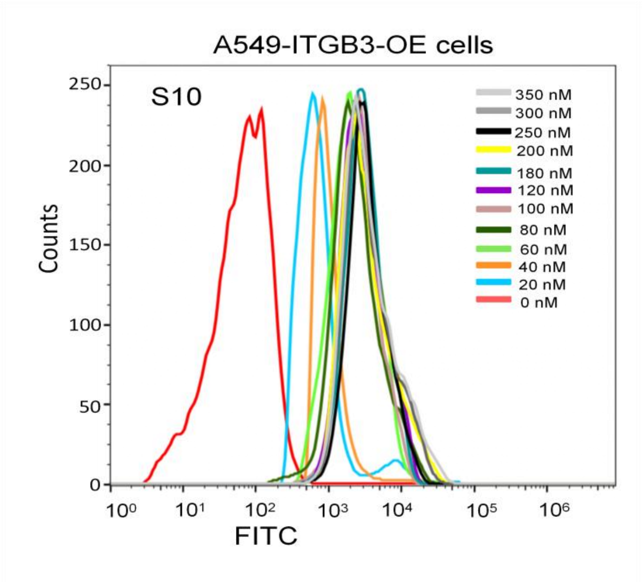


**Figure S4.** Monitoring the binding curves of FAM-S10 aptamer to A549-ITGB3-OE cells by FACS.

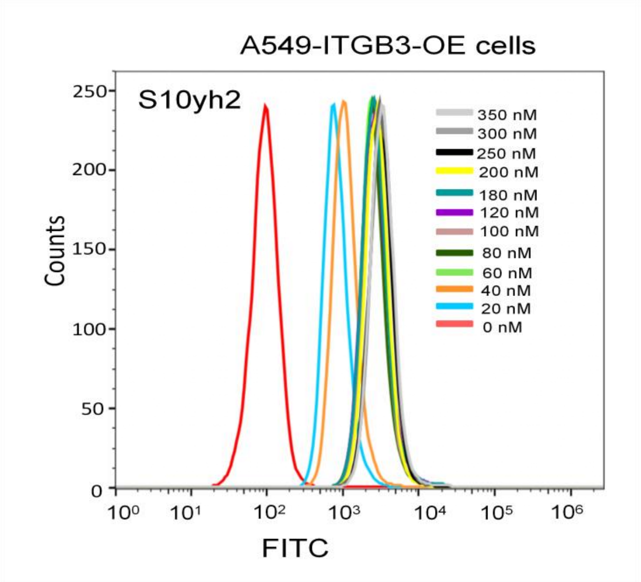


**Figure S5.** Monitoring the binding curves of FAM-S10yh2 aptamer to A549-ITGB3-OE cells by FACS.

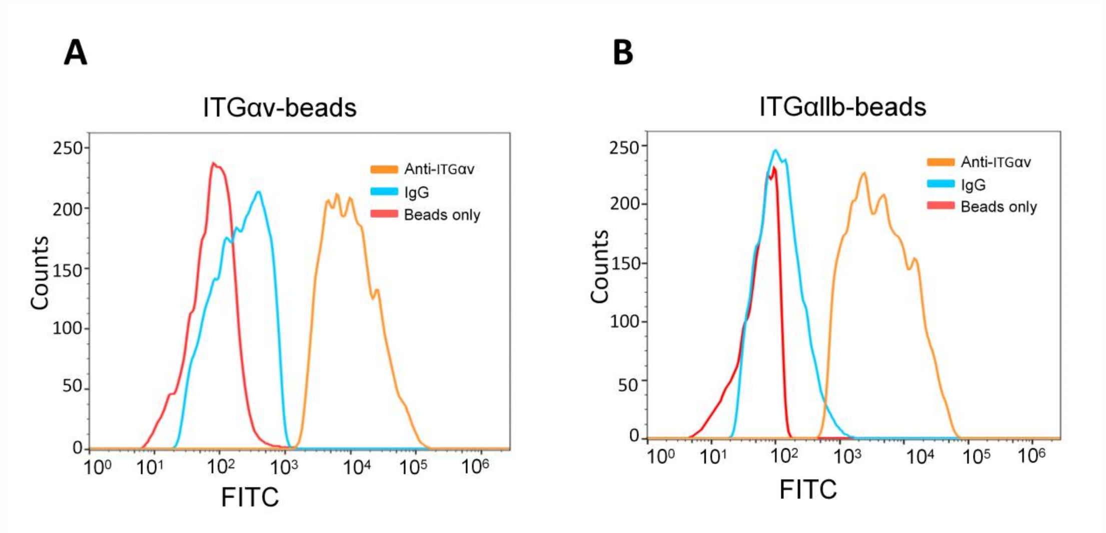


**Figure S6.** (A) Flow cytometry assay of beads costained by FITC-anti-ITGαv.
(B) Flow cytometry assay of beads costained by FITC-anti-ITGαllb.

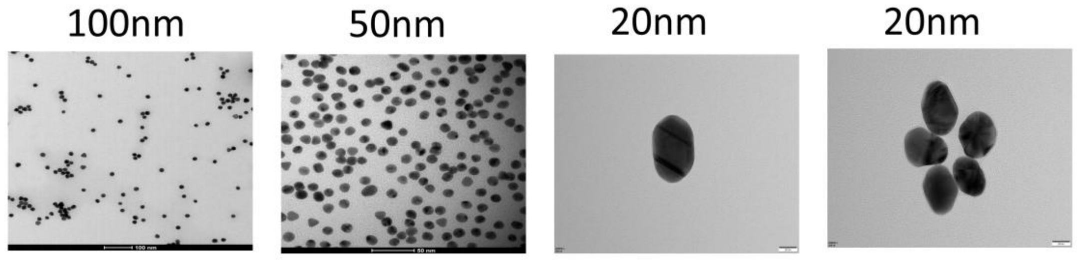


**Figure S7.** Electron Microscopy Image of Au Nanoparticles.
